# Multiparametric Characterization of the DSL-6A/C1 Pancreatic Cancer Model in Rats

**DOI:** 10.3390/cancers16081535

**Published:** 2024-04-17

**Authors:** Patrick Schmidt, Johannes Lindemeyer, Pranali Raut, Markus Schütz, Sven Saniternik, Jannika Jönsson, Heike Endepols, Thomas Fischer, Alexander Quaas, Hans Anton Schlößer, Martin Thelen, Holger Grüll

**Affiliations:** 1 Faculty of Medicine and University Hospital of Cologne, Institute of Diagnostic and Interventional Radiology, University of Cologne, 50937 Cologne, Germany; patrick.schmidt@uk-koeln.de (P.S.); johannes.lindemeyer@uk-koeln.de (J.L.); p.raut@erasmusmc.nl (P.R.); markus.b.schuetz@googlemail.com (M.S.); sven.saniternik@uk-koeln.de (S.S.); jannika.joensson@uk-koeln.de (J.J.); 2 Faculty of Mathematics and Natural Sciences, Department of Chemistry, University of Cologne, 50937 Cologne, Germany; 3 Faculty of Medicine and University Hospital of Cologne, Institute of Radiochemistry and Experimental Molecular Imaging, University of Cologne, 50937 Cologne, Germany; heike.endepols@uk-koeln.de; 4 Faculty of Medicine and University Hospital of Cologne, Department of Nuclear Medicine, University of Cologne, 50937 Cologne, Germany; thomas.fischer@uk-koeln.de; 5 Nuclear Chemistry, Institute of Neuroscience and Medicine (INM-5), Forschungszentrum Jülich GmbH, 52425 Jülich, Germany; 6 Faculty of Medicine and University Hospital of Cologne, Institute of Pathology, University of Cologne, 50937 Cologne, Germany; alexander.quaas@uk-koeln.de; 7 Center for Molecular Medicine Cologne, Faculty of Medicine and University Hospital Cologne, University of Cologne, 50937 Cologne, Germany; hans.schloesser@uk-koeln.de (H.A.S.); martin.thelen@uk-koeln.de (M.T.); 8 Department of General, Visceral, Cancer and Transplantation Surgery, Faculty of Medicine and University Hospital Cologne, University of Cologne, 50937 Cologne, Germany

**Keywords:** pancreatic cancer, DSL-6A/C1, magnetic resonance imaging, FAPI, cancer-associated fibroblasts, murine tumor model

## Abstract

**Simple Summary:**

In this study, a rat model of pancreatic ductal adenocarcinoma (PDAC) was established and extensively analyzed. The histological examination confirmed that the model displayed features of PDAC, such as malformed malignant ductal cells and cancer-associated fibroblasts (CAFs). The study delved into the tumor microenvironment, revealing a unique immune profile with increased CD8^+^ cytotoxic T cells, suggesting a stronger anti-tumor immune response. Different imaging techniques were employed, showing contrast enhancement on MRI and a strong uptake of ^68^Ga-FAPI (fibroblast activation protein inhibitor) tracers in tumor tissues. This model’s suitability for further research in ^68^Ga-FAPI imaging and therapeutic approaches was highlighted. While some limitations exist, such as late-stage edema and heterogeneous growth patterns, this model provides valuable insights for PDAC studies and potential therapeutic strategies.

**Abstract:**

The DSL-6A/C1 murine pancreatic ductal adenocarcinoma (PDAC) tumor model was established in Lewis rats and characterized through a comprehensive multiparametric analysis to compare it to other preclinical tumor models and explore potential diagnostic and therapeutical targets. DSL-6A/C1 tumors were histologically analyzed to elucidate PDAC features. The tumor microenvironment was studied for immune cell prevalence. Multiparametric MRI and PET imaging were utilized to characterize tumors, and ^68^Ga-FAPI-46-targeting cancer-associated fibroblasts (CAFs), were used to validate the histological findings. The histology confirmed typical PDAC characteristics, such as malformed pancreatic ductal malignant cells and CAFs. Distinct immune landscapes were identified, revealing an increased presence of CD8^+^ T cells and a decreased CD4^+^ T cell fraction within the tumor microenvironment. PET imaging with ^68^Ga-FAPI tracers exhibited strong tracer uptake in tumor tissues. The MRI parameters indicated increasing intralesional necrosis over time and elevated contrast media uptake in vital tumor areas. We have demonstrated that the DSL-6A/C1 tumor model, particularly due to its high tumorigenicity, tumor size, and ^68^Ga-FAPI-46 sensitivity, is a suitable alternative to established small animal models for many forms of preclinical analyses and therapeutic studies of PDAC.

## 1. Introduction

Pancreatic ductal adenocarcinoma (PDAC) is one of the most aggressive cancer entities, causing about 130,000 deaths in Europe per year and 4.2% of cancer-related deaths worldwide [1,2]. While the mean time of survival for other types of cancer has improved over the past years thanks to new therapeutic options, the 5-year survival rate for PDAC has remained low at 6% [3,4]. Due to a lack of symptoms and the early onset of metastases, PDAC is commonly diagnosed in a vast majority of patients at a late stage. The tumor tissue of PDAC is poorly perfused and characterized by a dense stroma with a high density of cancer-associated fibroblasts (CAFs). Poor drug penetration is therefore one of the reasons for the therapy resistance and poor response of PDAC [5,6,7].

Clinical diagnoses and staging are currently performed with Computed Tomography (CT) and Magnetic Resonance Imaging (MRI) [8]. CT is currently the primary imaging modality for initial diagnosis with an evaluation of the contrast agent uptake in the arterial and portal venous phase. Due to low perfusion and dense stroma, PDAC nodules appear to be hypointense in the arterial phase after contrast injection compared to the surrounding pancreatic tissue. The proper detection of small lesions or lesions that appear to be iso-dense to the surrounding tissue is challenging [9,10,11]. In this context, we recently showed that new energy-resolving CT systems, which provide for example iodine concentration maps, offer improved diagnostics of PDAC [12]. Magnetic Resonance Imaging (MRI) offers high-contrast soft tissue images based on different contrast mechanisms, such as T_1_ and T_2_, T_2_* weighted images, quantitative T_1_ and T_2_ maps, [13,14,15], diffusion-weighted images (DWIs) [16], perfusion, or dynamic contrast-enhanced images, allowing us to assess pharmacokinetic parameters [17,18]. Hence, MRI is an essential tool to gain in-depth knowledge about tissue properties and the tumor microenvironment and is suitable for monitoring therapy responses [19].

Due to the typically dense stroma in PDAC resulting from a high number of CAFs in the tumor tissue, imaging using Positron Emission Tomography (PET) tracers based on a ^68^Gallium (^68^Ga)-labelled fibroblast activation protein inhibitor (FAPI) has gained increasing importance in recent years [20]. Besides presenting a biomarker for tumor detection, CAFs are gaining increasing importance as targets of potential therapeutic options for PDAC due to their function as promoters of tumor growth, as well as immune suppression [21]. CAF imaging with ^68^Ga-FAPI PET/CT allows for improved detection of smaller lesions and an improved differentiation of PDAC from inflammatory processes compared to ^18^F-FDG-PET [22,23].

Research on new therapeutic options, as well as new imaging methods, for PDAC rely on a representative animal model mimicking the pathology that is observed in patients. For research purposes, a variety of animal pancreatic cancer models are known, mainly in mice. One of the most widely used models for PDAC is the genetically engineered KPC mouse, developed in 2005 [24]. The KPC model shows mutations in the KRAS and p53 genes that are typical for human PDAC, as well as a comparable dense stroma but lacks the typically high T cell density of human PDAC, limiting the use of the tumor model for immunological studies [25]. Another widely used PDAC tumor model is the murine syngeneic Panc02 cell line which is used for a wide range of immunobiological and immunotherapeutic analyses and therapeutic studies using chemotherapeutics and checkpoint inhibitors [26]. Although this tumor model shows a PDAC phenotype, it lacks a desmoplastic reaction, which is one of the main reasons for the weak sensitivity to chemotherapeutic agents in human patients [27]. In addition, xenograft tumor models from human PDAC represent the histological phenotype better than homograft animal models, but they also have various limitations, as immunosuppressed animals are used. Consequently, immune therapies, as well as the response of the immune system to therapies, are difficult to study. With respect to CAFs, xenograft models may form a different microenvironment in the tumor tissue, as murine fibroblasts are recruited from the surrounding host tissue and migrate into a human-derived tumor [28,29]. Furthermore, donor tissue can only be obtained from resectable human PDAC, for which only a minority of patients are eligible [30]. Moreover, as most of the established tumor models are grown in mice, their small tumor volume presents a challenge for performing therapeutic studies employing device-based physical therapies using radiofrequency, microwave, or high-intensity focused ultrasound [24,26,31]. Potential alternatives in larger animals for studies involving local therapies include neoplasms produced by carcinogenic agents in hamsters, some of which have high tumorigenicity, but show a very heterogeneous phenotype and are difficult to control, resulting in animals developing tumor entities in several organs besides the pancreas [32].

As a result of the above limitations, PDAC models in rats appear promising for studying certain therapeutic approaches which require larger tumor volumes or certain tissue and growth properties. In this study, we characterize the DSL-6A/C1 tumor model in detail, a pancreatic ductal cell line in the rat arising from a DSL-6 acinar cell carcinoma that was first described by Pettengill et al. in 1993 [33]. In particular, we use multiparametric MRI, FAPI-PET/CT, as well as histology and immunological and growth analysis of the DSL 6A/C1 PDAC tumor model to highlight its advantages and disadvantages compared to other tumor models.

## 2. Materials and Methods

### 2.1. Animals

Male Lewis rats were obtained from Charles River (Sulzfeld, Germany) and maintained in standard housing at 20–24 °C and 12 h light/12 h dark cycles. All animals were provided with water and food ad libitum. All animal experiments were conducted in accordance with local institutional guidelines and approved by the LANUV (Landesamt für Natur, Umwelt und Verbraucherschutz, Northrine-Westphalia, Germany).

### 2.2. Cell Line and Culture

The rat pancreatic ductal adenocarcinoma DSL 6A/C1 cell line derived from the DSL-6 acinar cell carcinoma was purchased from CLS (Cell Lines Service GmbH, Eppelheim, Germany) and cultured in Waymouth’s medium (Thermo Fisher Scientific, Schwerte (Northrine-Westphalia), Germany). The cell culture medium was supplemented with 10% heat-inactivated fetal bovine serum (FBS; Thermo Fisher Scientific), penicillin G (100 U/mL), and streptomycin (100 µg/mL). The cells were incubated at 37 °C in humidified air with 5% CO_2_. The medium was replaced twice weekly, and cells were maintained by serial passaging after trypsinization with Trypsin 0.5%/EDTA 0.2% in PBS.

### 2.3. Tumor Induction and Growth Measurement

For the creation of the tumors, male Lewis rats, 6–8 weeks of age, were anesthetized using a mixture of 3–5% isoflurane and oxygen. Meloxicam (0.5 mg/kg, s.c.) and metamizole (100 mg/kg, s.c.) were used as a pain medication. Then, 10^7^ DSL-6A/C1 cells were suspended in Matrigel and Waymouth medium (1:1, final volume 100 µL) and injected s.c. into each hindleg. Tumor dimensions were determined every second day by measuring the length (l), width (w), and depth (d) using a caliper, and the tumor volume was calculated using the formula V = 0.5 × l × w × d. In addition, tumor volumes were determined every second week using MRI with segmentations of T2-weighted images performed with ITK-SNAP [34]. In another group of 10 female Lewis rats, we did not observe any tumor growth over the time span of 5 months.

### 2.4. Euthanasia, Tissue Collection, and Histology

Euthanasia was performed in adherence to ethical standards and animal welfare considerations. The endpoint for each animal was determined by one or more of the following criteria: tumor size in one dimension exceeding 3 cm or manifestation of wasting syndrome. Some animals, especially in the slow-growing tumor group, had to be euthanized according to local COVID-19 restrictions. After euthanasia, tumors were extracted and fixed in 4% buffered formalin for 48 h prior to paraffin embedding. Sections were cut at 3 μm thickness. Representative sections were stained with Hematoxylin and Eosin (H&E) and analyzed for cancer-associated fibroblasts using a transmitted-light phase-contrast microscope (Zeiss Axioskop 2, Carl Zeiss, Jena (Thuringia), Germany).

### 2.5. Magnetic Resonance Imaging

After tumor cell injection, the animals underwent MRI monitoring every second week until euthanasia. The scans were acquired using a clinical 3.0 T MRI (Achieva^®^, Philips Healthcare, Best (North Brabant), The Netherlands) with a dedicated animal bed and ventilation setup embedded in an eight-channel receive-only rat body coil (RAPID Biomedical, Rimpar (Bavaria), Germany). The animals were anaesthetized with a mixture of isoflurane and oxygen before and during image acquisition, with the dose of isoflurane being adjusted to establish a therapeutically adequate anesthetic depth, while maintaining stable respiratory cycles. The hind legs of the animals were naturally cocked to the sides, resting between the lower abdomen and the side of the animal bed, close to the coil center and the magnet isocenter.

Tumors were localized with a transverse T_2_-weighted 2D turbo-spin echo (acronym T2TSE) sequence covering the animal roughly from the lower abdomen to the lower leg. Quantitative and contrast-enhanced acquisitions were recorded at selected time points (three time points are illustrated below). The following scans were obtained in the same reduced FOV of 60 × 60 × 21 mm^3^. For quantification of T_2_, a multi-slice turbo-spin echo sequence (acronym T2MAP) was acquired. Based on a Look–Locker-related acquisition (Ref Look–Locker), data for T_1_ relaxation mapping was obtained (acronym T1MAP) [35]. Dynamic contrast-enhanced (DCE) imaging was recorded based on a 3D FFE sequence. All relevant sequence parameters are listed in Table 1. Contrast agent (Clariscan^TM^, GE Healthcare, Chicago, IL, USA) was injected during the 15th dynamic acquisition using an automatic pump and an infusion line via the tail vein (0.2 mmol/kg bodyweight).

### 2.6. MRI Data Image Processing and Analysis

Based on the TSE2D data, the tumor volume was segmented for all subjects with ITK-Snap [34]. The T_2_ relaxivity was determined by fitting an exponential curve to the echo–time curve with non-linear least squares. For T_1_, the acquired data were flipped left of the zero-crossing and fitted to a simplified Look–Locker model, correcting for saturation effects as described in Deichmann et al. [36]. T_1_ and T_2_ maps, as well as the segmentation, were resampled to the through-slice resolution of the DCE data by nearest neighbor interpolation. In acquisitions where a hyperintense part within the tumor existed, it was segmented based on the resampled T_1_ maps. Image analysis for DCE data was performed with a dedicated open-source software, Rocketship [37], using a manually segmented vessel for AIF estimation and the provided nested model to derive the pharmacokinetic parameters. AIF smoothing and automated time point detection were employed.

### 2.7. Synthesis and Quality Control of ^68^Ga-FAPI

A total of 50 µg FAPI-46 (ABX, Radeberg, Germany) in 1.5 M HEPES buffer was labeled with 500 to 800 MBq [^68^Ga]-gallium chloride (^68^Ga-FAPI-46) in 0.1 M HCl using a ^68^Ge/^68^Ga generator (Eckert& Ziegler, Berlin, Germany) and the GRP synthesis module from Scintomics (Gräfelfing, Germany). The reaction was carried out at 125 °C for 10 min. Afterwards, the reaction mixture was purified via C18 column chromatography (Waters C18 cartridge) and consequently diluted with isotonic saline.

Radiochemical purity was determined by HPLC (Eluent A: water (HPLC Grade) + 0.1% trifluoro acetate (TFA), eluent B: acetonitrile (HPLC Grade) + 0.1% TFA, gradient: 95% A for 2 min, then to 5% A within 15 min, flow: 1.2 mL/min, UV detection: 254 nm, injection volume: 20 µL; free ^68^Ga was eluted after 2–3 min, product after 8 min) and TLC (ITLC-SG strips, 1.5 × 10 cm, 1 M Ammoniumacetat/Methanol 1:1, TLC Scanner from Raytest, Straubenhardt, Germany; ^68^Ga-colloid remained at the origin).

### 2.8. Positron Emission Tomography

Small animal PET imaging was performed when the tumor was visible on 3 consecutive MRI scans on a FOCUS 220 micro-PET scanner (CTI-Siemens, Germany). The rats were anesthetized with isoflurane in O_2_/air 3:7 (induction 5%, maintenance 2%), and a catheter for tracer injection was inserted into the lateral tail vein. After fixation in the animal holder, the emission scan started with intravenous injection of 10 to 64 MBq ^68^Ga-FAPI-46 in 500 µL 0.9% NaCl. The acquisition time was 60 min to 120 min. Emission scans were followed by an 8 min transmission scan with a rotating ^57^Co-point source.

Breathing rate and body temperature were monitored and held at approx. 60 breaths/min and 37 °C, respectively. The emission scans were histogrammed into time frames (2 × 1 min, 2 × 2 min, 6 × 4 min, 18 × 5 min for time–activity curves, and 4 × 30 min/4 × 15 min for display) and fully 3D rebinned (span 3, ring difference 47), followed by OSEM3D/MAP reconstruction. The resulting voxel sizes were 0.38 × 0.38 × 0.80 mm^3^. For all further processing of the images, including statistics, the software VINCI 5.21 for MacOS X (Max Planck Institute for Metabolism Research, Cologne, Germany) was used. Images were intensity-normalized to injected dose and corrected for body weight (SUV_bw_). To this end, every frame was divided by injected dose and multiplied by body weight times 100.

### 2.9. Isolation of Tumor-Infiltrating Lymphocytes from Rat Tissue and Flow Cytometry

Tumors used for immunological analysis had to be detectable on at least 3 consecutive MRI scans before removal. The scans took place every second week. During extraction, surrounding muscular tissue was removed. Tumor specimens were processed mechanically (gentleMACSDissociator, Miltenyi Biotech, Bergisch Gladbach (Northrine-Westphalia), Germany) and enzymatically (320 U/mL collagenase-IV, Worthington OH, USA and 100 U/mL DNAse-I, Applichem, Darmstadt (Hesse), Germany) at 37 °C for 1 h to obtain a single-cell suspension containing the tumor-infiltrating lymphocytes (TILs). The suspension was filtered using a 100 µm and a 70 µm cell strainer (Corning, New York, NY, USA). Spleens of tumor-bearing rats were mashed through a 100 µm cell strainer, followed by a subsequent filter step with a 70 µm cell strainer. Splenocytes and TILs were stained for multi-color flow cytometry (detailed antibody list, Appendix A Table A1). Intracellular FoxP3 staining was performed using the Foxp3/Transcription Factor Staining Buffer Set according to the manufacturer’s protocol (eBioscience, San Diego, CA, USA). Samples were acquired on a Cytoflex LX flow cytometer (Beckman Coulter, Brea, CA, USA).

**Figure 1:**
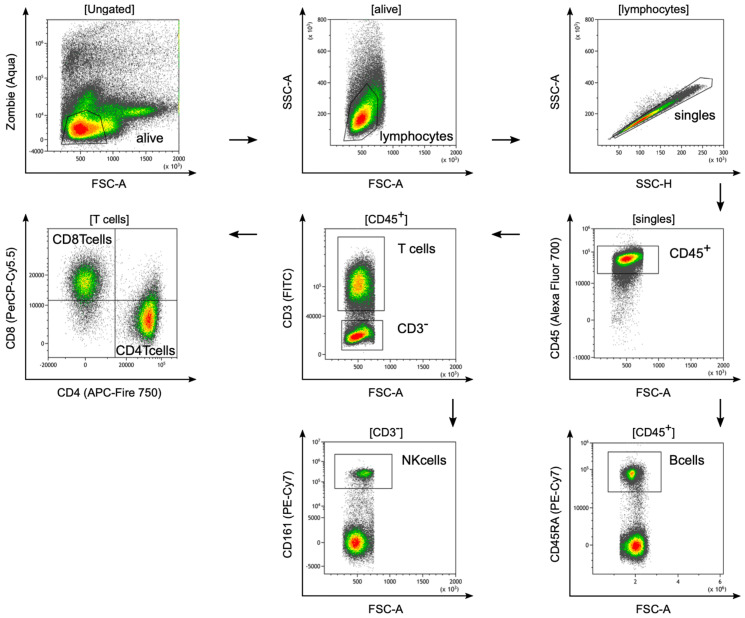
Representative flow cytometry plots and detailed gating strategy of tumor-infiltrating lymphocytes in DSL-6A/C1 tumors. Dead cells were excluded using a zombie dye (alive). Lymphocytes were selected by gating for size and granularity (Forward Scatter Area (FSC-A) versus Side Scatter Area (SSC-A)). Living lymphocytes were further plotted by SSC-H versus SSC-A to gate single cells. From the single cells (singles), lymphocytes (CD45^+^) were gated based on their CD45 expression. B cells were defined as CD45^+^ lymphocytes and the expression of CD45RA (B cells). T cells were defined by gating on CD45^+^ lymphocytes and the expression of CD3 (T cells), whereas CD3 cells expressing CD161 were considered NK cells (NK cells). T cells were further divided into CD8 T cells (CD8 T cells) or CD4 T cells (CD4 T cells).

To evaluate the potential of the pancreatic ductal adenocarcinoma cell line DSL-6A/C1 as a preclinical tumor model, TILs in DSL-6A/C1 tumors were analyzed. First, a single-cell suspension of fresh tumor tissue was generated after resection. Subsequently, lymphocyte subsets were analyzed by flow cytometry. Dead cells were excluded, and single lymphocytes were identified by forward–sideward scatter and CD45 expression. Subsequently, CD3^+^ T cells, CD3^+^CD4^+^ T cells and CD3^+^CD8^+^ T cells, CD19^+^ B cells, and CD3-CD56^+^ NK cells were identified, as depicted in Figure 1.

## 3. Results

### 3.1. Tumor Take-up Rate and Growth

**Figure 2:**
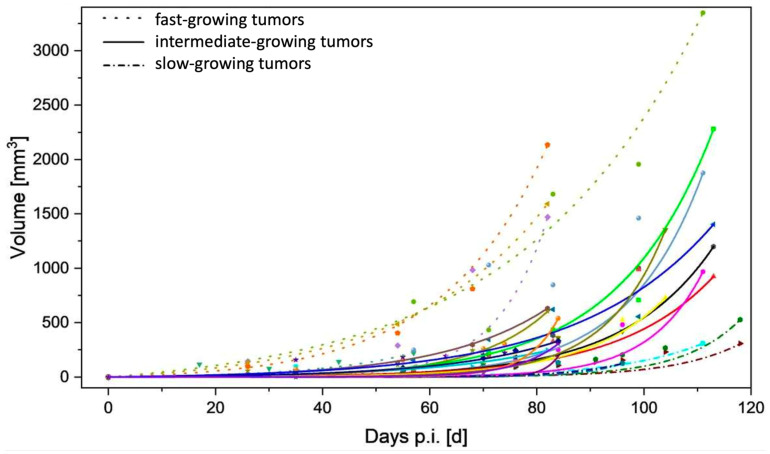
Tumor growth as a function of time. For a better overview, tumors were visually divided into fast-growing (dots only), intermediate-growing (solid lines), and slow-growing (dashed lines) tumors (p.i.—post injection). Missing time points reflect early euthanasia related to local COVID-19 restrictions.

**Figure 3:**
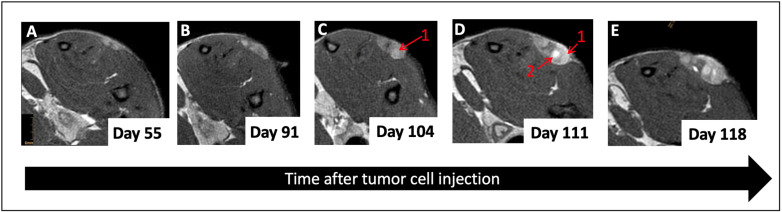
Representative T_2_ MRI images at various time points after inoculation. Unlike the earlier time points (**A**,**B**), the latest time points (**C**–**E**) show increasing edema (1) and cyst formation (2) within the tumor.

Subcutaneous tumor cell injections were successful in 16 out of 20 rats, resulting in a tumor formation rate of 80%, which is slightly lower than what is described in previous research [38]. In a group of 10 female rats, no tumor growth was seen over the time span of 5 months. Tumors were first identifiable (or visible) on MRI after 38 days (±12 days) and showed a very heterogeneous growth reaching an average tumor size of 815 mm^3^ (±628 mm^3^) after 100 days. Generally, all tumors demonstrated exponential growth after a variable time lag, varying from 40 days to 100 days after tumor implantation (Figure 2). In larger tumors, increasing intralesional edema and necrosis were seen over time and were clearly observable for 81% of all tumors on the last MRI scan (Figure 3), whereas distant metastases in the abdominal or thoracal cavity were not found either in PET-CT scans nor in autopsies. Cutaneous ulcerations were found in one out of twenty animals.

### 3.2. Multiparametric MRI

**Figure 4:**
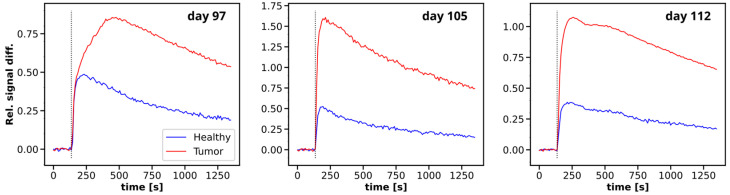
Average uptake curves of DCE acquisitions for tumor and healthy reference tissue on three different measurement days, illustrated by normalized difference to average baseline signal. Injection time is indicated by vertical black dashed line.

**Figure 5:**
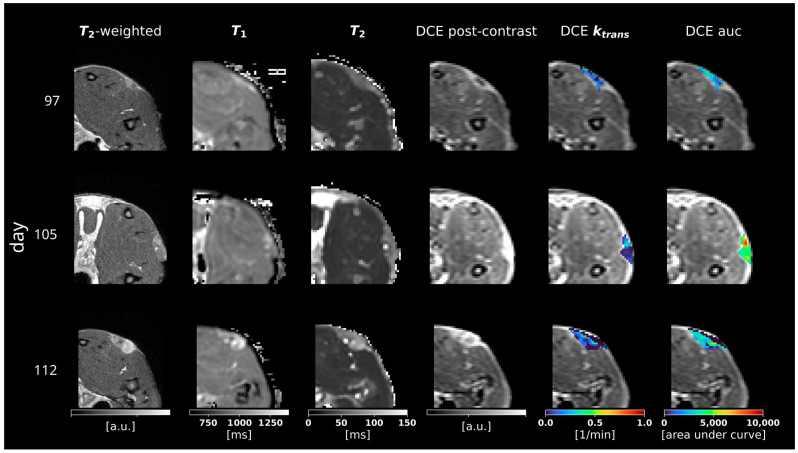
Representative slices of the acquired overview and quantitative MRI relaxation parameters, as well as the quantitative DCE MRI parameters (columns) at three different measurement days after inoculation (rows). The last three columns show the contrast-enhanced DCE magnitude as an underlay.

Examples for the acquired DCE signal magnitude curves are illustrated for three time points in Figure 4, while the calculated relaxation parameters can be found in Figure 5. The non-contrast T_1_ relaxation time in the tumor tissue showed moderately higher values compared to healthy muscle and remained at a constantly high level over the acquisition period. The tumors exhibited clear hyperintense contrast in the T_1_ maps, with an average difference of 80 to 250 ms (up to 1240 ms in tumors compared to 930 ms to 990 ms in tissue), which is comparable to other murine PDAC tumor models found in the literature [39,40].

The initial T_2_ values in the tumor tissue showed higher relaxation values compared to muscle (mean values of 60 to 90 ms in tumors compared to about 35 ms in muscles), which are slightly higher than those described in other small animal PDAC models [39]. Over time, the T_2_ relaxation times increased significantly in the central locations of the tumor and reached up to 110 ms, indicating the presence of cystic liquid formations within the lesion.

Due to the high perfusion and presence of malformed vascular structures within the PDAC tissue, the tumor showed significantly increased signal intensity after contrast agent injection in DCE images compared to healthy tissue, as displayed in Figure 5. As a measure of capillary permeability, the average tumor k_trans_ values ranged from 0.05 to 0.12 min^−1^, which is similar to other preclinical pancreatic cancer tumor models [41,42,43].

### 3.3. ^68^Ga-FAPI-46 PET Imaging

**Figure 6:**
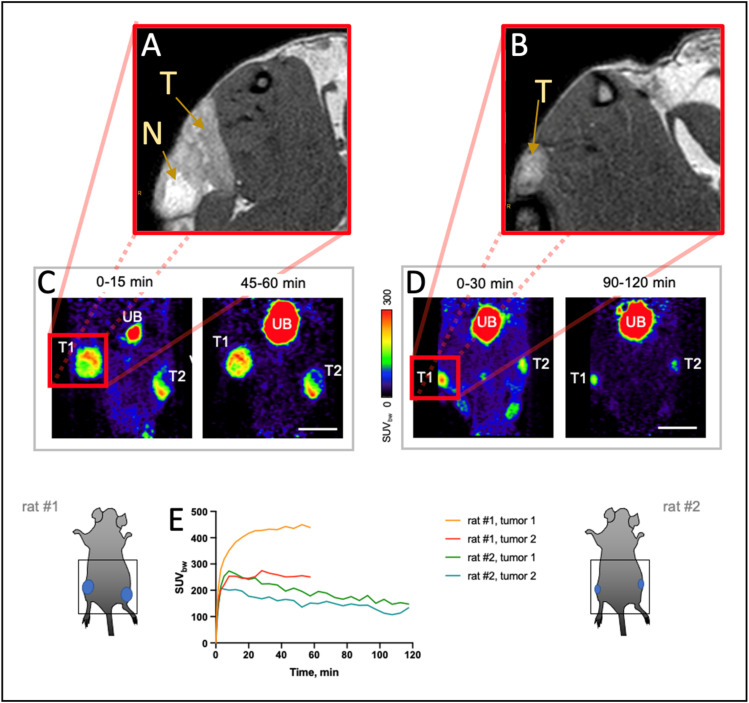
^68^Ga-FAPI-46 PET imaging of the DSL-6A/C1 tumors. (**A**,**B**) T_2_ MR images of the the s.c. tumors (T) at the time of PET acquisition showing central necrosis (N) in the larger tumors. (**C**–**E**) PET measurements with ^68^Ga-FAPI-46 in the same rats, each bearing two tumors; T1/T2—tumor 1/2; UB—urinary bladder; scale bar: 20 mm.

All tumors showed an immediate uptake of ^68^Ga-FAPI-46, which was higher in the bigger tumors of rat #1 compared to the small tumors of rat #2 (Figure 6). Other than the tumor tissue and the urinary bladder, through which the tracer is excreted, no other organ of the lower abdomen showed any notable uptake. The bigger tumors were heterogeneous, with a higher tracer uptake at the outer rim, while the center seemed to be necrotic. In the smaller tumors, the highest uptake was seen in the center.

### 3.4. Tissue Extraction and Histology

**Figure 7:**
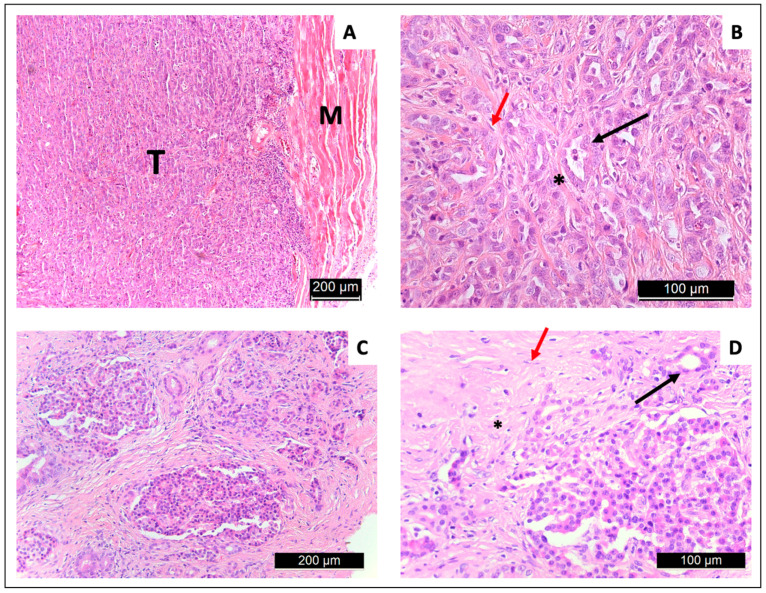
HE-stained histology slices of an s.c. DSL-6A/C1 neoplasm (**A**,**B**) in comparison to a human PDAC tumor (**C**,**D**), showing tumor (T) and muscle tissue (M), in the tumor stroma cancer-associated fibroblasts (red arrows) are present. Tumor cells forming tubulo-ductular structures (black arrows) and collagenous fibers (*) are found. Both carcinomas (rat and human) are formed by ductularly arranged tumor cells.

Macroscopically, solid and well-defined tumor tissue was present. This is histologically composed of abundant tumor cells that repeatedly form primitive ductular structures. In the peritumoral stroma, located interstitially among the tumor cells, CAFs were identified. All tumors showed a phenotype corresponding to ductal-type adenocarcinomas in HE-stained sections (Figure 7).

### 3.5. Tumor-Infiltrating Lymphocytes in DSL-6A/C1 Pancreatic Ductal Adenocarcinoma Tumors

**Figure 8:**
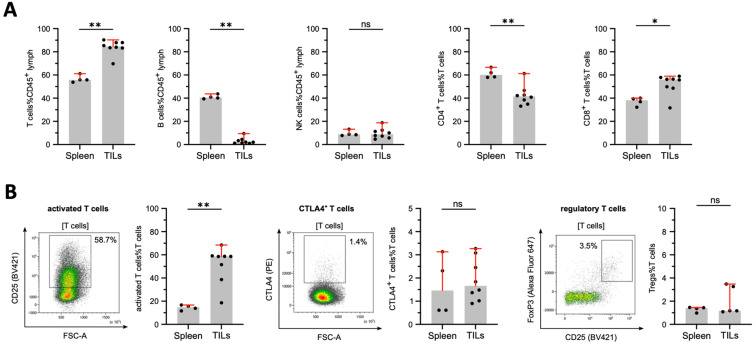
Tumor-infiltrating lymphocytes in DSL-6A/C1 tumors. (**A**) Lymphocyte subset in percent of all living lymphocytes. (**B**) Activated T cells (CD25^+^ T cells), regulatory T cells (CD4^+^CD25highFoxP3^+^ T cells), and CTLA4^+^ T cells in percent of all T cells. Significant differences, determined by the nonparametric, unpaired, two-tailed Mann-Whitney test, are denoted by asterisks (* *p* ≤ 0.05, ** *p* ≤ 0.01, ns: not significant). Mean ± 95% confidence interval is indicated in red where applicable.

TILs from DSL-6A/C1 tumors (left and right flank) with splenocytes of these rats (*n* = 4) were compared and showed an increase in CD3^+^ T cells in terms of percent of all CD45^+^ lymphocytes in TILs compared to splenocytes (Figure 8B). In the T cell fraction of TILs, an increased percentage of CD8^+^ cytotoxic T cells was found, whereas the percentage of the CD4^+^ T cell compartment was reduced in TILs compared to splenocytes. In addition, the flow cytometric analysis revealed that TILs contained fewer B cells, but similar percentages of NK cells compared with splenocytes (Figure 8B).

Next, functionally distinct subsets of these lymphocyte lineages based on specific phenotypes were analyzed and showed increased percentages of activated T cells in TILs, but similar percentages of regulatory T cells and similar expression levels of the co-inhibitory molecule CTLA-4 compared with splenocytes of tumor-bearing rats (Figure 1).

## 4. Discussion

The DSL-6A/C1 tumor cells were initially derived from an acinar cell carcinoma and displayed genetical characteristics of both acinar cell carcinoma and PDAC [44]. Our histological results, obtained from established tumors, showed features of PDAC, such as malformed pancreatic ductal malignant cells and CAFs in the tumor stroma [33,44]. Nevertheless, compared to human PDAC and tumor models in mice, the DSL-6A/C1 tumors showed a less pronounced deposition of collagen and desmoplastic stroma reaction [25,45]. The histology shows a more displacing growth pattern rather than infiltration of the surrounding tissue, which might be a result of the s.c. growth. A more infiltrative growth is described for this tumor model after orthotopic implantation in reference [38].

The tumor microenvironment plays a crucial role in cancer’s progression, metastasis, and response to therapies. Our findings highlight distinct immune landscapes in TILs compared to splenocytes. The increased presence of CD8^+^ cytotoxic T cells within the tumor microenvironment suggests an augmented anti-tumor immune response which, in human subjects, seems to be an indicator for longer survival [46]. In contrast, the observed decreased CD4^+^ T cell fraction raises questions about tumor-mediated immune alterations [47].

Interestingly, the reduced B cell prevalence in TILs might indicate a diminished role for humoral immunity in the tumor microenvironment, while the consistent NK cell percentages suggest unchanged innate immune surveillance. The elevated activated T cell percentages within TILs demonstrate the heightened immune reactions against tumor antigens. However, the parallel expression of regulatory T cells and the co-inhibitory molecule CTLA-4 between TILs and splenocytes hint at potential immune modulation within the tumor [48,49]. Comparatively, the immune profile of the DSL6-A/C1 tumor model exhibits a unique signature that is not commonly observed in other preclinical models, particularly in the context of CD8^+^ T cell infiltration [50]. Especially the relatively high inflammatory effector cells, such as NK cells and CD8^+^ T cells are, in humans, generally associated with longer survival and less aggressive tumor growth [51], which needs to be considered when using this tumor model for immunological studies on PDAC progression. Nevertheless, the balance between effector and regulatory elements within the DSL6-A/C1 model could offer valuable insights into the mechanisms of immune evasion, which are pivotal in the design of immunotherapies [52]. As flow cytometry was used as a method for in-depth characterization of TILs but with a lack of spatial resolution, a pronounced tumor rim location of the immune cells is possible and needs further investigation. Furthermore, the unique genetical characteristics, with a lack of the typical Tp53 and KRAS mutations and properties of both ductal and acinar pancreatic carcinoma, that is described in the literature need to be taken into account when further analyzing the immune signaling of the DSl-6A/C1 PDAC tumor model [44].

Tumors were further characterized with multimodal, multiparametric MRI and PET imaging. On MR scans, tumorous tissue produced clear contrast in multiple MRI parameters, such as an increased contrast on T_1_- and T_2_-weighted images. The T_1_ and T_2_ relaxation values are significantly higher compared to the surrounding tissue and in the range reported from other groups analyzing the DSL-6A/C1 tumor model and for other tumors induced in the pancreatic head [53,54]. The T_2_-weighted scans also revealed increasing intralesional edema, observable in almost all tumors at later time points, which is a consequence of a central necrosis. The latter is typically observed at the late stages in poorly perfused malignant tumors. In contrast to the typical hypointense human PDAC, DCE MR acquisitions of s.c. DSl-6A/C1 tumors show an increase in contrast agent enhancement at the tumor rim and, in the early stages, within the tumor compared to healthy tissue. A possible explanation is the subcutaneous localization that might contribute to an atypical vascularization pattern, which is divergent from orthotopic tumors. Moreover, a maintained localized inflammatory response could also account for the observed peripheral enhancement in the later stages of tumor progression. These findings need further investigation in future studies but might underscore the importance of tumors’ microenvironment and location in mediating vascular dynamics and may have implications for the interpretation of imaging results and the design of therapeutic strategies. However, poorly perfused intralesional areas associated with central necrosis are found in later stages of tumor progression. The relatively dense tumor stroma is also reflected by low k_trans_ values derived from DCE-MR measurements. The k_trans_ values are considerably lower compared to human PDAC [55], but similar compared to other fast-growing murine subcutaneous tumor models [41,43].

PET imaging using ^68^Ga-FAPI tracers demonstrated strong uptake in all tumor tissues, with low off-target uptake of the tracer and urinary excretion. The tracer binds specifically to the FAP protein, which is overexpressed on CAFs. Our histological findings confirm the presence of CAFs in the tumor microenvironment, which may explain the significant uptake of tracers within the tumor, along with the high sensitivity of PET imaging. However, recent studies also showed that FAP can be overexpressed on cancer cells themselves, among others also in human-derived pancreatic cancer cell lines [56,57,58]. So far, the FAP expression of DSL-6A/C1 has not been investigated, but it will be the topic of a future study. Furthermore, ^68^Ga-FAPI uptake can not only be seen in CAF, but also in non-malignant conditions, such as inflammatory lymph nodes, artherosclerosis, or surgical scars [59]. Nevertheless, the high tracer uptake is described in ^68^Ga-FAPI PET images obtained from human PDAC patients [22,60]. CAFs have been identified as a target in tumor diagnosis and therapy, as they seem to play a key role in various parts of tumor development, such as tumor growth, metastasis formation, and the immune response [61,62]. Clinically, ^68^Ga-FAPI-PET imaging can be used to screen for distant metastases, but also to evaluate therapy efficacy and to optimize staging in the context of therapy planning [22,23].

No local or distant metastases were detected by post-mortem workup of the organs and on ^68^Ga-FAPI-PET. Notably, also no local lymph node metastases were found in the iliac and inguinal stromal areas. However, distant metastases to lymph nodes and the liver have been described in the literature for this tumor model [54], but since these occurred in tumors that grew orthotopically in the pancreas, the reason may be a consequence of our protocol, where tumors were implanted subcutaneously in the hind legs.

Generally, subcutaneous DSL-6A/C1 tumors show a heterogenous growth pattern, with exponential growth after a variable and sometimes relatively long time lag ranging from 40 to 100 days after implantation. Nevertheless, the average tumor volume of 815 mm^3^ after 100 days is significantly larger compared to the tumor volumes which can be achieved in in mice. This model is therefore particularly advantageous for studying certain physical therapeutic interventions using devices such as radiofrequency applicators or magnetic resonance-guided high-intensity focused ultrasound (MR-HIFU) [63]. Furthermore, the slow-growing tumors together with the high uptake of FAP-specific tracers offer the possibility to study different PET tracers in consecutive imaging studies within the same animal. Therefore, less animals are needed for tracer development, and the results are less affected by inter-animal variations or rapid tumor growth, which is often associated with large changes in the uptake pattern.

Limiting factors in terms of the usability of the DSL-6A/C1 model are the frequent formation of intralesional edema in the late stages of tumor growth and the heterogeneous growth pattern, resulting in a relatively unpredictable treatment window for interventional therapies. Also, the subcutaneous models may show differences in their microenvironment and metastasis formation compared to an orthotopic tumor implantation, which has not yet been investigated. In female rats, no tumor growth was observed due to unknown reasons.

## 5. Conclusions

In summary, we have demonstrated in initial experiments that the DSL-6A/C1 tumor model, particularly due to its high tumorigenicity, ^68^Ga-FAPI sensitivity, and tumor size, is a suitable alternative to established small animal models for many forms of preclinical analysis and therapeutic studies of PDAC.

## Figures and Tables

**Table 1:** Summary of MRI scanning parameters.

Protocol	TSE2D	FFE2D	T2MAP	T1MAP	DCE
Sequence Type	2D TSE	2D FFE	2D TSE	IR Look–Locker	3D FFE
TE (ms)	30	4.8	6.25 + *n* × 25	3.7	2.8
TR (ms)	12117	230	11970	8000	6
Field of View (mm^3^)	~90 × 60 × 50	~90 × 60 × 66	60 × 60 × 21	60 × 60 × 21	60 × 60 × 20
Reconstruction voxel size (mm^3^)	0.2 × 0.2 × 1.0	0.28 × 0.28 × 1.00	0.63 × 0.63 × 2.1	0.63 × 0.63 × 2.1	0.62 × 0.62 × 0.7
Acquisition voxel size (mm^3^)	0.3 × 0.3 × 1	0.3 × 0.3 × 1	0.94 × 0.94 × 2.1	0.94 × 1.00 × 2.1	0.7 × 0.7 × 0.7
NSA	1	2			
Flip Angle (°)	90	60	90	5	15
No. of frames (Slices)	40	60	10	10	29
Fat saturation	no	no	SPIR	SPIR	no
Other parameters				TI = 22 + *n* × 100 ms	RF-spoiled, dyn. scan time = 9.7 s

Abbreviations: TSE: Turbo Spin Echo; FFE: Fast Field Echo; IR: Inversion Recovery; *n*: echo number [0, …, 15] for T2MAP and [0, …, 64] for T1MAP, respectively; SPIR: Spectral Presaturation with Inversion Recovery; RF: Radiofrequency.

## Data Availability

The data presented in this study are available in the article.

## Appendix A

**Table A1:** List of antibodies used for the immunological analysis of the DSL-6A/C1 tumors.

Antibody	Fluorochrome	Clone	Host	Company	Catalogue
Zombie Aqua				Biolegend	423102
CD38	FITC	REA683	Human cell line	Miltenyi	130-110-277
CD3	FITC	REA223	Human cell line	Miltenyi	130-102-678
IgG	PerCp Cy5.5	Poly4054	Goat	Biolegend	405424
CD8a	PerCP-Vio 700	REA437	Human cell line	Miltenyi	130-132-092
CD54	PE	1A29	Mouse	Biolegend	202405
CD11a	PE	REA596	Human cell line	Miltenyi	130-109-171
CD152 (CTLA4)	PE	WKH203	Mouse	Biolegend	203007
CD27	PE Dazzle 594	LG.3A10	Armenian Hamster	Biolegend	124228
CD45RA	PE-Cy7	OX-33	Mouse	Biolegend	202316
CD161	PE-Vio 770	REA227	Human cell line	Miltenyi	130-102-714
CD86	APC	24F	Mouse	Miltenyi	130-109-130
CD314 (NKG2D)	APC	REA471	Human cell line	Miltenyi	130-106-992
FoxP3	Alexa Fluor 647	150D	Mouse	Biolegend	320014
CD45	Alexa Fluor 700	OX-1	Mouse	Biolegend	202218
CD4	APC Cy7	W3/25	Mouse	Biolegend	201518
CD80	BV421	3H5	Mouse	BD	743863
CD25	BV421	OX-39	Mouse	BD	565608
CD134 (OX40)	BV421	OX-40	Mouse	BD	744815
